# Thalamocortical seizure onset patterns in drug-resistant focal epilepsy

**DOI:** 10.1093/braincomms/fcag227

**Published:** 2026-06-19

**Authors:** Hugh D Simpson, Vaclav Kremen, Vladimir Sladky, Benjamin H Brinkmann, Nicholas M Gregg, Brian N Lundstrom, Kai J Miller, Jamie J Van Gompel, Gregory A Worrell

**Affiliations:** Department of Neurology, Mayo Clinic, Rochester, MN 55905, USA; Department of Neurology, The Alfred Hospital, Melbourne, VIC 3004, Australia; Department of Neuroscience, Monash University, Melbourne, VIC 3004, Australia; Department of Neurology, Mayo Clinic, Rochester, MN 55905, USA; Czech Institute of Informatics, Robotics, and Cybernetics, Czech Technical University in Prague, Prague 16000, Czech Republic; Department of Natural Sciences, Faculty of Biomedical Engineering, Czech Technical University in Prague, Kladno 27201, Czech Republic; Department of Neurology, Mayo Clinic, Rochester, MN 55905, USA; Czech Institute of Informatics, Robotics, and Cybernetics, Czech Technical University in Prague, Prague 16000, Czech Republic; Department of Natural Sciences, Faculty of Biomedical Engineering, Czech Technical University in Prague, Kladno 27201, Czech Republic; Department of Neurology, Mayo Clinic, Rochester, MN 55905, USA; Department of Neurology, Mayo Clinic, Rochester, MN 55905, USA; Department of Neurology, Mayo Clinic, Rochester, MN 55905, USA; Department of Neurosurgery, Mayo Clinic, Rochester, MN 55905, USA; Department of Neurosurgery, Mayo Clinic, Rochester, MN 55905, USA; Department of Neurology, Mayo Clinic, Rochester, MN 55905, USA

**Keywords:** epilepsy, neurostimulation, thalamus, EEG, seizure

## Abstract

Drug-resistant epilepsy affects tens of millions of people worldwide and is associated with considerable morbidity and mortality. Thalamic deep brain stimulation and cortical responsive neurostimulation are proven treatments for focal epilepsy. Both have been used to target a range of thalamic nuclei; yet, the roles of these thalamic nuclei in focal seizure generation remain incompletely understood. Thirteen patients with drug-resistant focal epilepsy undergoing intracranial EEG were consented to undergo investigation of thalamocortical networks. Sampled regions included cortical, mesial temporal, and thalamic brain regions. Visual and spectral analyses were performed to identify seizure onset patterns and correlate thalamic and cortical seizure activity. Thalamic ictal discharges were observed in 89% of seizures. Of these, 56% demonstrated synchronous thalamocortical activity with distinct patterns. These onset patterns included hypersynchronous spiking, low-voltage fast activity, ictal baseline shifts, and broadband suppression. Multiple thalamic nuclei were involved in ictal organization and propagation, with the specific nuclei depending on the cortical seizure network. The thalamus plays a crucial role in focal onset seizure generation and propagation, with distinct seizure onset patterns and nuclei involved. These findings support exploring a broader range of thalamic nuclei in epilepsy neurostimulation and have implications for seizure detection settings in intracranial sensing devices.

## Teaser

Simpson *et al*. report the results of an intracranial EEG study demonstrating synchronous involvement of the thalamus in focal seizures, in distinct patterns that parallel those seen in cortex, with specific thalamic nuclei corresponding to cortical onset regions. These findings have implications for neurostimulation target selection and seizure detection algorithms.

## Graphical Abstract

**Graphical Abstract:**
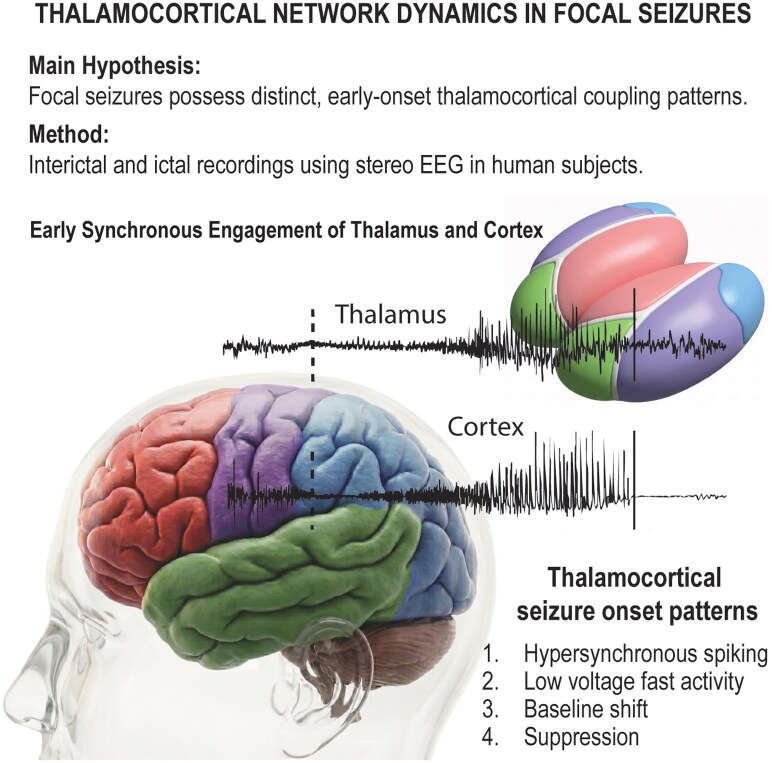

## Introduction

Drug-resistant epilepsy (DRE) affects up to one-third of individuals with epilepsy worldwide and is associated with substantial morbidity and mortality.^1^ While resective or ablative surgery remains the most effective treatment for appropriately selected patients with focal epilepsy, many are poor candidates for such interventions, while some who undergo surgery may yet continue to experience seizures. For these individuals, electrical brain stimulation (EBS) offers a promising adjunctive therapy.

EBS modalities, including deep brain stimulation (DBS), responsive neurostimulation (RNS) and vagus nerve stimulation (VNS), are effective in reducing seizure frequency, with median reductions of up to 75%.^2-4^ Additionally, some patients achieve seizure freedom for months to years. Among these techniques, DBS of the anterior nucleus of the thalamus (ANT) has Class I evidence for efficacy in focal epilepsy.^2,3^ Other thalamic nuclei, such as the centromedian nucleus (CMT), show promise for generalized and multifocal epilepsy.^5,6^ Emerging evidence also highlights the potential of pulvinar nucleus (PUL) DBS in focal epilepsy, though its mechanisms remain underexplored.^7-9^ Despite these advances, significant gaps persist in our understanding of thalamocortical networks and their role in seizure generation (ictogenesis) and propagation. Moreover, recent studies challenge traditional assumptions about thalamocortical circuitry, such as the surprising efficacy of ANT-DBS in generalized epilepsy,^10^ and the use of CMT-DBS in focal epilepsy.^11^ Consequently, many facets of EBS, including optimal patient selection, target choice for specific epilepsy syndromes and stimulation parameters, remain poorly defined.

The role of the thalamus in ictogenesis and epileptogenesis is a topic of continued interest.^8,12-14^ Intracranial local field potential (LFP) recordings suggest that various thalamic nuclei—including the ANT, mediodorsal thalamus (DMT) and PUL—are recruited during seizure onset and propagation.^15,16^ This is of particular relevance for programming accurate seizure detectors using sensing-capable DBS devices^17^ and RNS systems. While originally used exclusively for cortical seizure detection and responsive stimulation in hippocampus and neocortex, there is growing interest in targeting thalamic nodes with RNS.^7^ Thus, the timing and patterns of thalamic involvement in ictogenesis become crucial for programming detectors. Often, seizure detection is cortically based, while stimulation may be cortical and/or thalamic. This is based on the assumption that cortex leads thalamus in seizure onset in focal epilepsy^18^ as opposed to the simultaneous thalamocortical dynamics seen in some generalized epilepsies.^19^ The possibility of synchronous thalamocortical networks in focal epilepsy would challenge this dogma and approach. Finally, while cortical seizure onset patterns are well characterized,^13,20^ thalamocortical network correlates of these patterns is only beginning to be understood.^21^

In this study, we aim to advance our understanding of the thalamus’s role in ictogenesis by analysing thalamocortical network dynamics during focal seizures.

## Materials and methods

### Study population

Thalamocortical seizure onsets were studied in 13 patients with drug-resistant focal epilepsy. Two groups were included. The first was made up of 10 patients undergoing stereo-electroencephalography (sEEG) as part of routine presurgical evaluation, who had a single electrode trajectory extended to sample the thalamus, where it was safe and feasible to do so. This was performed under Institutional Review Board (IRB) ethical approval (IRB 15-007984). The second group was made up of three patients implanted with an investigational neural sensing and stimulation device (RC + S^TM^) with bilateral ANT and mesial temporal leads; under FDA Investigational Device Exemption (G180224) and IRB approval (IRB 18-005483). All participants provided informed consent. Clinical characteristics and outcomes were reviewed retrospectively.

### Data analysis

Seizure onset and propagation patterns were analysed visually and quantitatively using custom MATLAB scripts. In the RC + S^TM^ group, automatic seizure detection was used to identify candidate seizures first, as described previously.^22^ In both groups seizures were confirmed using visual review by two trained epileptologists and a consensus onset time label created independently for the cortical an thalamus LFP. Thalamocortical ictal circuits were characterized based on the timing and spectral patterns of thalamic discharges relative to cortical SOZ activity. Descriptive statistics were used for analysing EEG seizure characteristics, including thalamocortical delays and amplitudes. Due to the delays and amplitudes being non-normally distributed, the Mann–Whitney U test (with an alpha of 0.05) was used for group comparisons. Non-normality was established with formal testing (Anderson-Darling test) and visual inspection of Q-Q plots.

Preoperative 3T MRI was co-registered with postoperative CT scans for localization of electrode contacts. Lead DBS was used to identify thalamic nuclei.^23^ Groupings of thalamic nuclei included the following: anterior (ANT: anteroventral, anteromedial, anterodorsal), ventral (ventral anterior, ventral lateral, ventral posterior), intralaminar (centromedian, centrolateral, parafascicular), medial (mediodorsal) and posterior (pulvinar, lateral posterior).

## Results

### Patient and seizure characteristics

**Figure 1:**
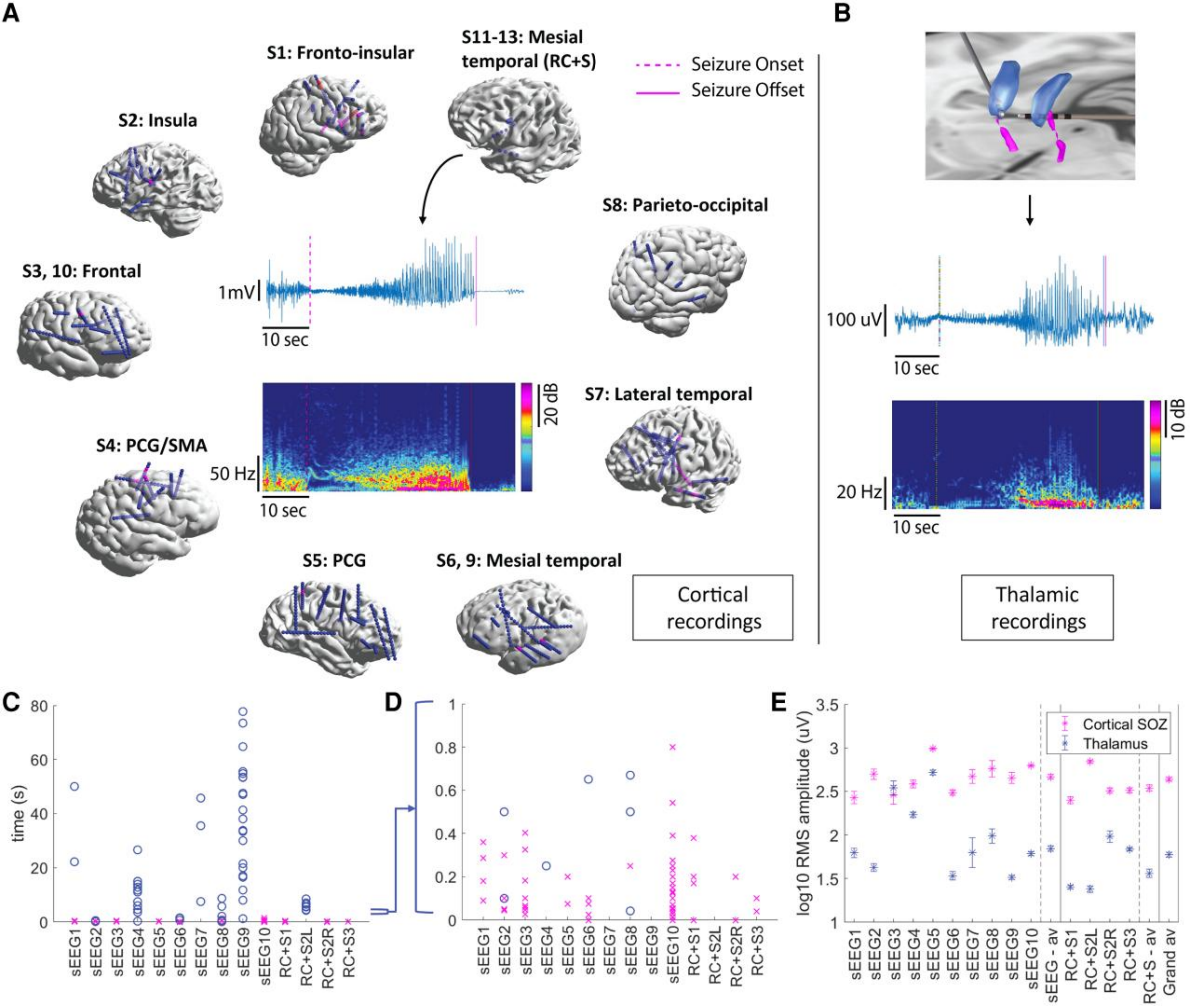
**Simultaneous cortical and thalamic ictal onset recordings, corticothalamic onset delays and ictal amplitude comparison**. (**A**) 3D reconstructions of stereo-electroencephalography (sEEG) and chronic ambulatory device electrode implantations surrounding an example of the raw EEG recording from an exemplar cortical seizure onset zone (hippocampus; *top*), as well as time-frequency analysis of the cortical seizure onset zone discharge (bottom). PCG, precentral gyrus; SMA, supplementary motor area. **(B**) *Top*—3D reconstruction of a thalamic electrode implantation targeting anterior thalamic nuclei bilaterally, parcellated and coloured individually according to the Morel atlas.^30^ Blue—anteroventral (AV) nucleus; magenta—mammillothalamic tract. Middle/bottom—example bipolar EEG recording from the same anterior nucleus of the thalamus (AV; middle) with time-frequency analysis of the thalamic ictal discharge (bottom). The raw EEG and time-frequency plots in **A** and **B** depict recordings from a single seizure in a single participant. Dashed lines = seizure onset, solid lines = seizure offset; magenta = cortical discharge, green = thalamic discharge. (**C** and **D**) Individual corticothalamic ictal onset delays are plotted for each seizure for each subject, sEEG1-10 and RC + S^TM^ 1–3. All delays are shown in **C**, while **D** shows only the 0–1 s range. (**E**) Log 10 transformed root mean square (RMS) voltage amplitude of ictal discharge in cortical seizure onset zone (SOZ; in magenta) and thalamus (blue) are averaged and plotted for each participant, and averaged again across the two groups (sEEG and RC + S^TM^; error bars are standard error of the mean). In **C** and **D**, each column represents a single subject (sEEG1 is the first sEEG subject, and so on) with the exception of RC + S2, for which each hemisphere is represented separately (RC + S2L = left hemisphere, RC + S2R = right hemisphere); and each marker (‘o’ or ‘x’) represents a single seizure. In **E**, the same notation applies as **C** and **D**, with the addition of: ‘sEEG—av’ denoting the average of the 10 sEEG subjects, ‘RC + S—av’ denoting the average of the 3 sEEG subjects, and ‘Grand av’ denoting the average of all 13 subjects.

Thirteen patients were included in the study: 10 from the sEEG group and 3 from the RC + S^TM^ group (Fig. 1). The median age of participants was 33 years (range: 20–65 years) (Supplementary Table 1). There were no complications of thalamic electrode implantation in either group. Across both groups, 158 seizures were analysed (Supplementary Table 2).

In the sEEG group, six patients had extra-temporal SOZ, with one case of multifocal extra-temporal onset. Two patients had temporal lobe SOZ, and two had multifocal temporal and extra-temporal SOZ. Thalamic sampling in this group included anterior (2/10), medial (4/10), intralaminar (6/10), ventral (6/10) and posterior (3/10) nuclei.

In the investigational device group, two patients had unilateral mesial temporal involvement in recorded seizures, while one patient exhibited bilateral mesial temporal seizure involvement. All seizures in this group were associated with anterior thalamic electrode recordings.

Across all patients, distinct thalamic ictal discharges were observed in at least one seizure. Of the 158 seizures analysed, 140 (89%) demonstrated thalamic ictal discharges. Among these, 79 (56%) exhibited thalamocortical primary organization patterns, while the remainder were propagation patterns.

Corticothalamic delays ranged from near simultaneous (0–50 ms) to prolonged (60–90 s) (Fig. 1C and D; Supplementary Fig. 1). The median delay for primary onset patterns was 53 ms [interquartile range (IQR) 0–182 ms], while for propagation patterns the median was 11.7 s (IQR 4.5–33.8 s); and these delays were significantly different (Mann–Whitney *U* = 3345, *P* < 0.001).

The amplitudes of electrographic discharges in the thalamus were significantly lower than in the cortex, by almost an order of magnitude (Fig. 1E; Supplementary Fig. 2). The median root mean square (RMS) voltage in the thalamus was 57 µV (IQR 30–84 µV) as compared to 408 µV (IQR 283–635 µV) in the cortex (Mann–Whitney *U* = 33924, *P* < 0.001).

### Thalamocortical seizure onset patterns

**Figure 2:**
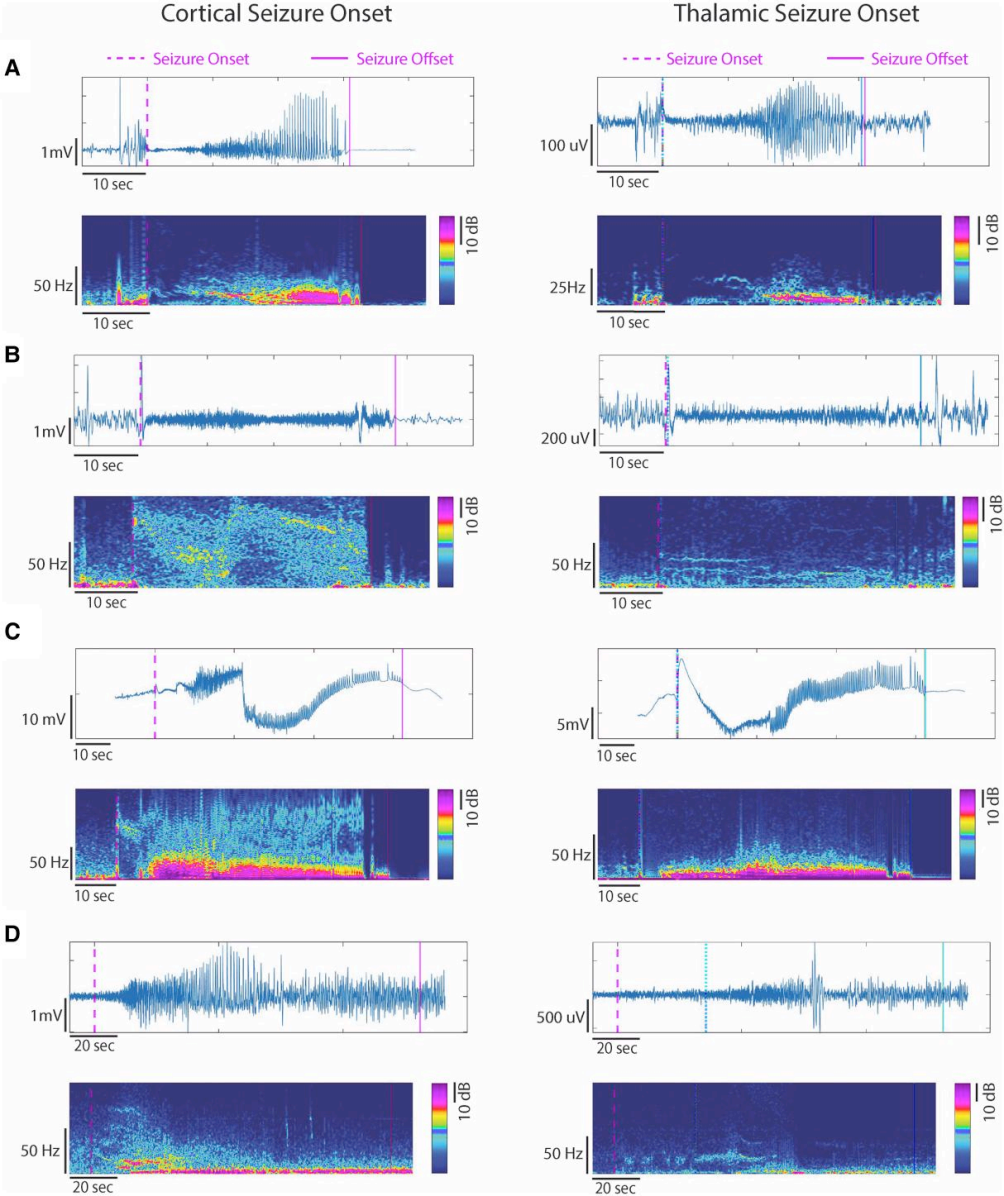
**Corticothalamic propagation and seizure onset patterns**. Four examples of corticothalamic patterns are shown—one seizure from each of four different subjects is shown in each of **A**, **B**, **C** and **D**. All panels: top row = raw EEG, *bottom* row is time-frequency plot (spectrogram); left column = cortical EEG zone, right column = thalamic EEG. Dashed lines = seizure onset, solid lines = seizure offset; magenta = cortical discharge, green = thalamic discharge. (**A**) Seizure onset in the mesial temporal region is essentially synchronous with the thalamus (anterior nucleus), with pre-ictal spiking followed by low-frequency suppression and low voltage fast activity seen in both mesial temporal and thalamic regions. (**B**) Seizure onset in the posterior insula with synchronous thalamic discharge, consisting of hypersynchrony (large spike) followed by low frequency suppression and low-voltage fast activity. The same progression—pre-ictal spiking, ictal baseline shift, and low voltage fast activity (LVFA) is seen in the ventral thalamus (ventral lateral nucleus) albeit with slightly different spectro-temporal characteristics to the cortex. (**C**): seizure onset in the dorsolateral frontal lobe (middle frontal gyrus) is synchronous with thalamic onset (mediodorsal nucleus), the latter manifested as a large ictal baseline shift/slow wave with marked suppression of other frequencies, and eventually hypersynchrony in the form of repetitive spiking (no high pass filter in **C**, to allow for viewing of slow wave). (**D**): seizure onset occurs in the lateral temporal region, while thalamic activity (ventral posterior lateral nucleus) is delayed by over 30 s; representing a propagation pattern.

Distinct thalamocortical patterns were observed, with seizure onsets classified as synchronous/early or delayed (Fig. 2). Four characteristic thalamic onset patterns were identified, and listed along with how frequently they were observed (see also Supplementary Tables 2 and 3):

1. Hypersynchronous activity: Pre-ictal periodic or repetitive spiking, or sentinel spikes followed by seizure onset; 5/13 subjects.
2. Low-voltage fast activity: High-frequency LFP activity (>25 Hz); 7/13 subjects.
3. Slow wave/Ictal baseline shift: A prominent LFP slow wave or baseline shift at onset; 3/13 subjects.
4. Suppression: Broad suppression of thalamic LFP background activity; 8/13 subjects.

These patterns could occur in isolation or in combination. For example, hypersynchronous activity was frequently followed by low-voltage fast activity (LVFA), while baseline shifts often coincided with suppression of baseline LFP activity. Cortical and thalamic patterns were sometimes near identical but could also be distinct. For instance, LVFA in the cortex was often higher in frequency (50–150 Hz) and descended in frequency with time, compared to the corresponding thalamic LVFA (30–50 Hz), which often remained static with time. LVFA and suppression were the most common patterns (7/13 and 8/13 subjects, respectively) and were seen together in 7/13 subjects. When present, LVFA, suppression and baseline shifts were the most consistent patterns within subjects, while the presence of hypersynchrony was more variable (Supplementary Table 3). These patterns also demonstrated variation in the anatomical location in which they were observed in the thalamus (Supplementary Table 3). Ictal baseline shifts were the most restricted, seen only in medial or ventral thalamus; while hypersynchronous activity was seen in anterior, ventral and intralaminar nuclei; and LVFA and suppression were seen in all regions.

Thalamocortical primary organization patterns were specific to the thalamus (not observed in non-thalamic non-seizure onset zone channels) and stable under differing montages (Supplementary Figs 3 and 4).

### Thalamocortical propagation and termination patterns

Propagation patterns were characterized by delayed thalamic ictal discharges, often observed 10–60 s after cortical seizure onset (Fig. 1C; Fig. 2D; Supplementary Fig. 1). These delayed discharges were associated with rhythmic ictal LFP oscillations (1–20 Hz) in thalamic contacts. In rare cases (4/13 patients; 5/158 seizures), thalamic discharges persisted beyond cortical seizure offset, and in one case, the thalamic discharge appeared to precede obvious cortical activity (Supplementary Fig. 5).

### Organization of thalamic ictal recruitment in space and time

**Figure 3:**
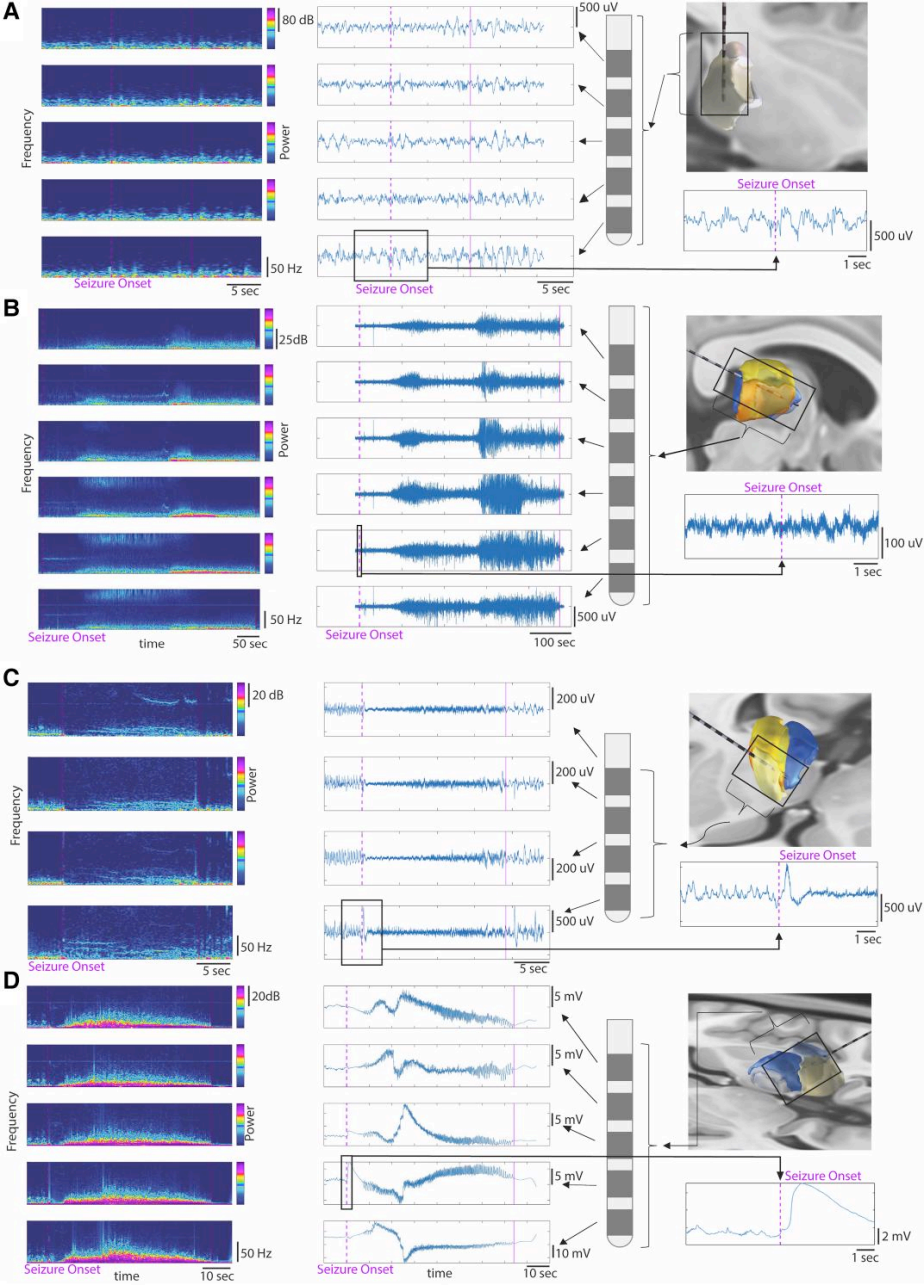
**Thalamic ictal recruitment patterns**. Four examples of different thalamic recruitment patterns are shown—one seizure from each of four different subjects is shown in each of **A**, **B**, **C** and D. (**A–D**) All panels, left to right: time-frequency plot (left); raw EEG (middle); schematic of stereo-electroencephalography (sEEG) electrode with most distal contact at bottom of image, anatomical location of electrode, and zoomed in version of raw EEG (5 s before and after cortical seizure onset); (right). For each **A–D**: rows represent different bipolar recording channels in sampled thalamus. Dashed lines = seizure onset, solid lines = seizure offset. Different thalamic recruitment patterns are shown: (**A**) no definite thalamic involvement; (**B**) long period of focal-appearing activity (∼40 Hz in spectrogram) in a thalamic propagation pattern before more diffuse recruitment; (**C**) a focal sentinel sharp wave at onset with maximal amplitude (Contacts 1 and 2), followed by well organised low voltage fast activity primarily restricted same contacts as evident on the spectrogram, before a diffuse suppression of background frequencies, with evolution of recruitment of different thalamic regions over time; and (**D**) a focal, large amplitude slow wave with phase reversal (contacts 1–2), reflecting a focal thalamus direct current (DC)/baseline shift change at seizure onset followed by more ‘diffuse’ thalamic (no high pass filter in **D**, to allow for viewing of slow wave).

Thalamic LFP activity during seizures varied across patients and seizure types but was stereotyped for the same pattern of seizures within individual patients. Regional thalamic activation varied from no activation (Fig. 3A), to focal-appearing activity (Fig. 3B and C), to more mixed focal and diffuse changes (Fig. 3D). Broadband or low frequency suppression of thalamic LFP background rhythms was commonly present diffusely throughout the thalamus, often with superimposed more focal patterns (e.g. Figure 3C). Distinct spectral patterns could be observed to be spatially restricted (e.g. 1–2 electrode contacts), often correlated with a specific thalamic nucleus (e.g. ANT); and vary through time during a seizure.

### Thalamocortical seizure circuits

**Figure 4:**
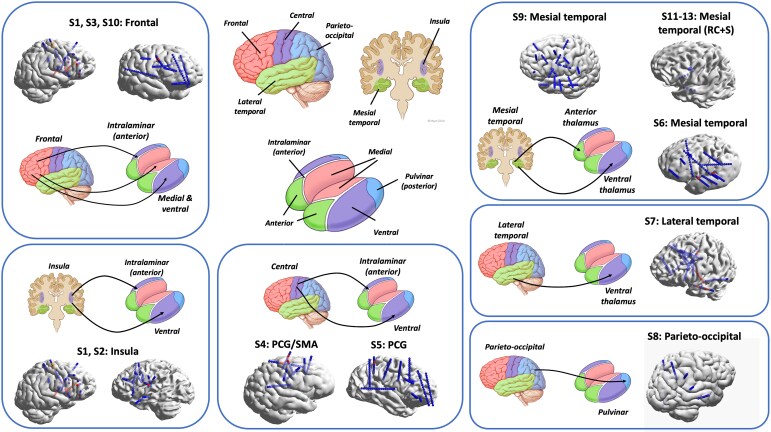
**Corticothalamic seizure circuits**. Circuits are grouped according to seizure onset zone and earliest thalamic recruitment. Clockwise from *top* left: frontal to intra-laminar, medial, and ventral thalamus; insula to intralaminar and ventral groups; central/peri-rolandic to intralaminar/ventral groups; parieto-occipital to posterior (pulvinar) group; lateral temporal to ventral group; mesial temporal to anterior/ventral groups. For each circuit, electrode positions [stereo-encephalography (sEEG) or RC + S^TM^] are shown in 3D reconstructed models, while circuit schematics depicting functional connectivity based on ictal EEG data are shown by arrows. PCG, precentral gyrus; SMA, supplementary motor area.

The location of initial focal ictal activity within the thalamus varied according to the cortical SOZ, allowing for functional connectivity to be inferred from thalamocortical ictal activity (see Supplementary Methods for details). Characteristic thalamocortical seizure circuits were identified (Fig. 4), including:

- Frontal SOZ: connected to medial, ventral and intralaminar thalamus.
- Insular SOZ: connected to ventral and intralaminar thalamus.
- Central/peri-rolandic SOZ: connected to ventral and intralaminar thalamus.
- Parieto-occipital SOZ: connected to posterior thalamus.
- Lateral temporal SOZ: connected to ventral thalamus.
- Mesial temporal SOZ: connected to anterior and ventral thalamus.

These circuits are largely consistent with known structural and functional thalamocortical networks, including the more diffuse projections associated with intralaminar nuclei.

## Discussion

Our findings highlight the central role of the thalamus in focal epilepsy, with ictal discharges observed in multiple thalamic nuclei across the majority of seizures. We observed thalamocortical networks at seizure onset characterized by thalamic activity that was simultaneous with cortical seizure onset and often paralleled this activity, suggesting a more intricate and complex thalamocortical dynamics underlying focal ictogenesis than is often assumed. More specifically, we identified primary organization patterns—characterized by distinctive synchronous (or near synchronous) thalamic discharges—and propagation patterns, in which thalamic activity followed cortical seizure onset by seconds to minutes. The involvement of diverse thalamic nuclei—including from the anterior, ventral, medial, posterior and intralaminar groups—and multiple cortical connections of each nucleus, underscore the thalamus’s broad functional connectivity.

Distinct ictal onset patterns in the thalamus—hypersynchronous spiking, LVFA, ictal baseline shift and suppression—were analogous to previously described cortical patterns,^20^ though with lower amplitudes and differing spectral characteristics. This suggests that the thalamic generators are closely coupled with cortical seizure activity while contributing unique dynamics to ictogenesis and propagation.

Similarly, we noted that thalamic ictal activity can be focal or diffuse and can evolve in space and time within intrinsic thalamocortical networks. This is analogous to these same well-described patterns in cortical seizure onset and propagation.

### Implications for neurostimulation therapies

Current DBS targets, such as the ANT and CMT, have been selected based on their connectivity to frontal and temporal networks and demonstrated efficacy in clinical trials.^2,4,5^ Our results suggest that other thalamic nuclei, including the pulvinar, medial and other intralaminar thalamic nuclei, may also play key roles in focal epilepsy. Expanding DBS targets to include these regions, or combining stimulation at multiple thalamic nodes, could enhance therapeutic efficacy by suppressing onsets and propagation. Notably, we observed distinct thalamocortical circuits corresponding to different cortical SOZs. Tailoring stimulation targets based on individual thalamocortical networks may improve seizure control and reduce variability in patient outcomes. In addition, the classification of thalamic activity as focal or diffuse may confer prognostic information and inform the choice of focal thalamic stimulation (e.g. ANT-DBS) versus other forms of neurostimulation (e.g. VNS), similar to the way focality in SOZ influences decision-making in resective or ablative surgery.

Corticothalamic RNS approaches, which are being increasingly explored, require choosing electrodes for detection and stimulation. One strategy is to use a cortical lead for detection, given their presumed earlier role in focal seizure onset, while using a thalamic lead for stimulation. Our work suggests that appropriately placed thalamic leads could be used for both detection and stimulation, and the specific onset patterns we have identified could be used to choose and tune detectors for more accurate and timely seizure detection. This agrees and extends our other findings with RC + S^TM^ devices published by Gregg *et al.*^17^

### Implications for epilepsy diagnostics

The robust and stereotyped nature of thalamic ictal patterns raises the possibility that thalamic electrodes could serve as ‘sentinel’ electrodes during sEEG evaluations, for example, by lateralizing the SOZ in rapidly propagating seizure networks, or—as appeared to be the case in one seizure in one subject in our analysis—identifying unsampled cortical regions contributing to seizure networks.

The differences in LFP amplitude and power spectral density observed in thalamus and cortex is related to the structural organization of the thalamus and biophysics properties of closed fields versus the open field of laminar structure in the cortex.^24^ The cortical columnar and laminar architecture generates higher amplitudes through summation, whereas the spherical/radial cellular nature of thalamic nuclei results in some cancellation of electric fields, and thus lower amplitudes and spectral power. The practical implication of this is that care must be taken in reading sEEG on standard sensitivities/gain, as low amplitude thalamic discharges may be missed. Similarly, removing high pass filters can allow for better visualization of ictal baseline shifts, and spectral analysis can be a valuable aid if available.

In addition to guiding patient selection and tailoring neurostimulation therapies through passive recordings, trial stimulation of thalamic targets during sEEG may help predict responses to chronic stimulation, further optimizing target selection for individual patients.^25^

Finally, there were no complications of thalamic sEEG implantation in this study, adding to the growing body of work supporting the safety of this procedure.^26^

### Pathophysiological insights

This study reinforces the thalamus’s role as a hub in thalamocortical networks, dynamically linking cortical regions during ictogenesis and propagation. Mechanistically, the simultaneous and highly correlated nature of cortical and thalamic activity at focal seizure onset challenges the notion of the cortex as an independent focal seizure generator, at least in some cases. In generalized onset seizures, while undoubtedly seizures ‘start’ somewhere, the specific location probably varies, and is less important than existence of the underlying diffuse hyperexcitable network. In the same way, it may make less sense to talk about focal seizures ‘starting’ in the cortex, and instead preferable to consider hyperexcitable thalamocortical networks, which may range from very focal to more diffuse.

### Comparison with similar work

Our findings are comparable to those of Wu *et al.,*^12^ who found the first thalamic region activated during seizures to be variable, and not always correspond to classical thalamocortical circuits, in contrast to our results. Follow-up work also found an unexpectedly important role for PUL compared to ANT in temporal lobe seizures.^14^ Related analyses have demonstrated variability in thalamic involvement related to cortical onset pattern and focality, though with more prolonged thalamocortical delays than we observed^27^; and additionally the involvement of the mediodorsal nucleus in temporal lobe epilepsy, with otherwise similar intra-thalamic spatial evaluation to what we observed.^21^ Thalamic sampling in paediatric populations has demonstrated both a similar synchronous onset to what we observed,^28^ as well as a similar range of onset patterns,^29^ though additionally describing rhythmic slowing and not observing ictal baseline shifts or suppression.

### Limitations

Generalizability of our findings is limited by the relatively small sample size and retrospective nature of the study. Thalamic nuclei were also not sampled systematically as some other studies have done and the absence of surgical outcomes in most patients precludes definitive confirmation of cortical SOZ localization. Hence, inferences around functional connectivity based on our data and therefore conclusions of our seizure circuit analysis, remain guarded and need to be evaluated with more systematic studies. It is also important to note that anatomical localization of thalamic contacts is challenging due to the small size and close proximity of nuclei, limiting precision in this regard, and some analyses (e.g. exploring the effects of alternative montages on findings) were only possible using the sEEG data, and not with the more limited sampling and recording capability of the implantable RC + S^TM^ system.

### Conclusion

This study highlights the critical role of the thalamus in the organization and propagation of focal seizures, providing insights into thalamocortical network dynamics in focal epilepsy. Our work contributes to the growing body of evidence supporting a network-based approach to epilepsy management and paves the way for novel diagnostic and therapeutic innovations.

## Supplementary Material

fcag227_Supplementary_Data

## Data Availability

The source data for the results presented in this manuscript are available from the authors on reasonable request. Code used to analyse and visualize the data is available at: https://github.com/hdsimpson/tsr-analysis-2026.
