# Supplementary material for: Thalamocortical seizure onset patterns in drug-resistant focal epilepsy
Source: Brain Commun. 2026 Jun 19;8(4):fcag227. doi: 10.1093/braincomms/fcag227 (PMC13326942; doi:10.1093/braincomms/fcag227)
Supplement: fcag227_Supplementary_Data [file fcag227_supplementary_data.pdf]

Supplementary Material for: Thalamocortical seizure onset patterns in drug resistant focal epilepsy

Supplementary Methods

Patient characteristics

Patients were implanted with stereo-encephalography (sEEG) between October 2016 and March 2022; and with the investigational RC+S^{TM} device, between, November 2019 and April 2021. All patients were followed up from implantation until 25 November 2024.
The median age was 33 years (range: 20-65), the median follow-up time was 41 months (range 1-87 months), and the median seizure reduction at end of follow-up was 81% (range: 20-99%) (Supplementary Table 1).

sEEG Recordings

Prolonged sEEG recordings were acquired from the 10 patients as part of their routine inpatient clinical evaluation for drug resistant focal epilepsy. Lead electrode placements were determined by a multidisciplinary team of epileptologists, neuroradiologists, and neurosurgeons, targeting hypothesized seizure onset zones (SOZ). Stereo-EEG recordings were acquired using a Natus Xltek or Quantum system at a sampling rate of 256–500 Hz and filters 0.05 – 100 Hz.

Over the course of this study, our center completed 223 intracranial EEG evaluations for drug-resistant focal epilepsy. Of these, 37 were subdural grid and strip implantations and 186 were sEEG cases. Of the 186 sEEG cases, 15 included thalamic sampling. The first 10 of these thalamic sampling cases were performed under the IRB-approved research protocol focused on assessing safety, feasibility, and potential clinical relevance. Subsequently, and for the next five patients who underwent thalamic sEEG sampling in the study period, thalamic sampling was based on clinical decision-making and recommendations from our multidisciplinary epilepsy surgery conference. The current analysis includes only data from the 10 patients who were consented under the IRB research protocol.

As described in the main text, in the 10 patients studied under IRB, trajectories were extended into the thalamus to allow sparse sampling of thalamocortical networks involved in seizure generation and propagation. The densely packed nature of thalamic nuclei typically

resulted in more than one nucleus being sampled in these single passes. However, given the anatomical arrangement of nuclei within the thalamus, and typical sEEG trajectories used, certain combinations of nuclei (e.g. ANT and PUL) were not possible to sample simultaneously.

Investigational Device Recordings

Three patients with mesial temporal lobe epilepsy were implanted with an investigational system (RC+S^{TM}) with rechargeable battery and continuous LFP streaming and recording. Each patient had bilateral leads targeting amygdala/hippocampus and ANT. Signals were recorded at 250Hz sampling rate and filtered to (0.85-100 Hz band). Data were recorded over multiple months using a bipolar montage, and streamed to a cloud-based server via a tablet device. Seizures were automatically detected and confirmed through visual review by trained epileptologists as described previously.[1]

Neurophysiologic data analysis

Continuous local field potential (LFP) recordings were notch-filtered at 60 Hz, and additional high-pass filtering (>0.5 Hz) was applied (except where noted in relevant figures). Cortical and thalamic ictal discharges were classified based on time-domain and spectral features, including root mean square (RMS) amplitude and power-in-band analysis for delta (0–4 Hz), theta (4–8 Hz), alpha (8–13 Hz), beta (13–30 Hz), and gamma (30–100 Hz) frequency bands.

Corticothalamic delays were qualitatively analysed independently by two experienced EEG reviewers (HS and GW) who agreed on the onset times for seizure discharges in the cortex and thalamus. The latency between the cortical onset and thalamus was determined using an expanded time-scale of 1-second for visual review. For seizure onset (or primary organisation) patterns, time of onset was defined as the first definite EEG change that evolved according to standard EEG criteria (evaluation in frequency, amplitude, spatial extent). For propagation patterns in the thalamus, the onset was defined as the first definite change from the background but not fulfilling the above ictal criteria.

Thalamic seizure onset patterns were categorised into primary organisation patterns and propagation (non-specific) patterns. Primary thalamocortical organisation patterns were defined as EEG patterns observable in the thalamus simultaneously (or near simultaneously)

with cortical seizure onset, that were distinct from interictal thalamic activity and analogous to known cortical seizure onset patterns in visual qualitative and quantitative spectral characteristics[2,3].

For each subject, cortical seizure onset zone is related to initial and maximal thalamic activation (Supplementary Table 2). Thalamocortical seizure circuits (Fig. 4, main manuscript) were then generated in the following manner: For each seizure that exhibited a thalamocortical primary onset pattern, a functional connection was inferred between the seizure onset zone (SOZ) for that seizure, and thalamic nucleus displaying the pattern. Where more than one nucleus displayed the pattern, the first nucleus was chosen, and when two or more nuclei were simultaneous, the most prominent nucleus (that with the highest amplitude and/or best organised rhythm) was chosen. When the thalamic pattern was diffuse or nuclei were indistinguishable based on the above, the functional connectivity inferred was for both nuclei. Functional connections were then collated and displayed graphically as arrows between functionally connected regions.

Descriptive statistics were calculated for seizure characteristics, thalamocortical delays, and power analysis.

Neuroimaging

Identification of specific thalamic nuclei was performed using Lead DBS and the Krauth/Morel atlas, adjusted for use with the Montreal Neurological Institute (MNI) 2009b asymmetric template space used in the Lead-DBS package.[4]

Supplementary Results

Corticothalamic ictal delays

Delays between cortical and thalamic onset for ictal discharges was generally consistent within subjects typically falling into either the synchronous or near synchronous category (Supplementary Fig 1A, Subjects 2, 3, 5, 10; Supplementary Fig 1B, Subjects 1, 2R, 3), or the delayed/propagation category (Supplementary Fig 1A; Subjects 1, 4, 7, 8, 9; Supplementary Fig 1B, Subject 2L). Some subjects demonstrated a mixture of both (Supplementary Fig 1A; Subjects 6 & 10).

Cortical and thalamic ictal power

Ictal EEG power varied significantly between cortex and thalamus (Supplementary Fig. 2). In most subjects across most frequency bands power in the cortex was higher than that in the thalamus, however in a few subjects there was little to no difference or the trend was reversed in the low (delta) frequencies (Supplementary Fig 2A, Subjects 3, 5)

Effects of alternative montages

For the sEEG participants, we explored alternative montages using a series of references including scalp (i.e. standard referential montage that was used for acquisition of the sEEG recordings), as well as digital re-referencing using distant white matter contacts and channels uninvolved in the epileptogenic zone based on visual review of the sEEG (Supplementary Fig. 3). Main results (onset patterns and delays) were reproducible in these montages, however certain artefacts (e.g. 60 Hz line noise and apparent volume conduction artefact) were occasionally observed, which were not seen in the bipolar montage we employed (Supplementary Fig. 3C & G). Amplitudes of thalamic EEG had some dependence on montage choice, but typically maintained order of magnitude difference with cortical amplitudes.

For the RC+S^{TM} participants, due to the nature of the recording device, only bipolar recordings were available for analysis, so the effects of alternative montages were not able to be explored.

Specificity of thalamocortical primary organisation patterns

For the sEEG participants, the specificity of the thalamocortical primary organisation patterns can be seen by comparing thalamic channels to the same epochs in non-thalamic non-seizure onset zone channels (Supplementary Fig. 4). The absence of the thalamocortical primary organisation patterns in these comparator channels demonstrates that these patterns are specific to the thalamus, and not due to volume conduction or choice of montage.

For the RC+S^{TM} participants, due to the nature of the recording device, additional channels were not available for analysis, so the specificity of primary organisations patterns to the thalamus was not able to be explored.

Thalamic discharges may precede cortical ictal discharges

In the vast majority of seizures with accompanying thalamic discharges, the thalamic activity was synchronous with or delayed from the cortical onset. We observed one example of an ictal thalamic discharge that appeared to precede the cortical activity by a few seconds (Supplementary Fig. 5). This was thought to represent ictal activity in unsampled cortex, rather than a focal onset in the thalamus. While the thalamic activity corresponded to a propagated discharge and not a primary organisation patter, it illustrates the principle that thalamic sampling can in principle detect seizures occurring in unsampled cortex.

Supplementary References

1. Sladky V, Nejedly P, Mivalt F, et al. Distributed brain co-processor for tracking spikes, seizures and behaviour during electrical brain stimulation. *Brain Communications*. 2022;4(3). doi:10.1093/BRAINCOMMS/FCAC115

2. Lagarde S, Buzori S, Trebuchon A, et al. The repertoire of seizure onset patterns in human focal epilepsies: Determinants and prognostic values. *Epilepsia*. 2019;60(1):85-95. doi:10.1111/EPI.14604

3. Pizzo F, Roehri N, Giusiano B, et al. The Ictal Signature of Thalamus and Basal Ganglia in Focal Epilepsy: A SEEG Study. *Neurology*. 2021;96(2):e280-e293. doi:10.1212/WNL.0000000000011003

4. Horn A, Kühn AA. Lead-DBS: A toolbox for deep brain stimulation electrode localizations and visualizations. *NeuroImage*. 2015;107:127-135. doi:10.1016/j.neuroimage.2014.12.002

Supplementary Figures

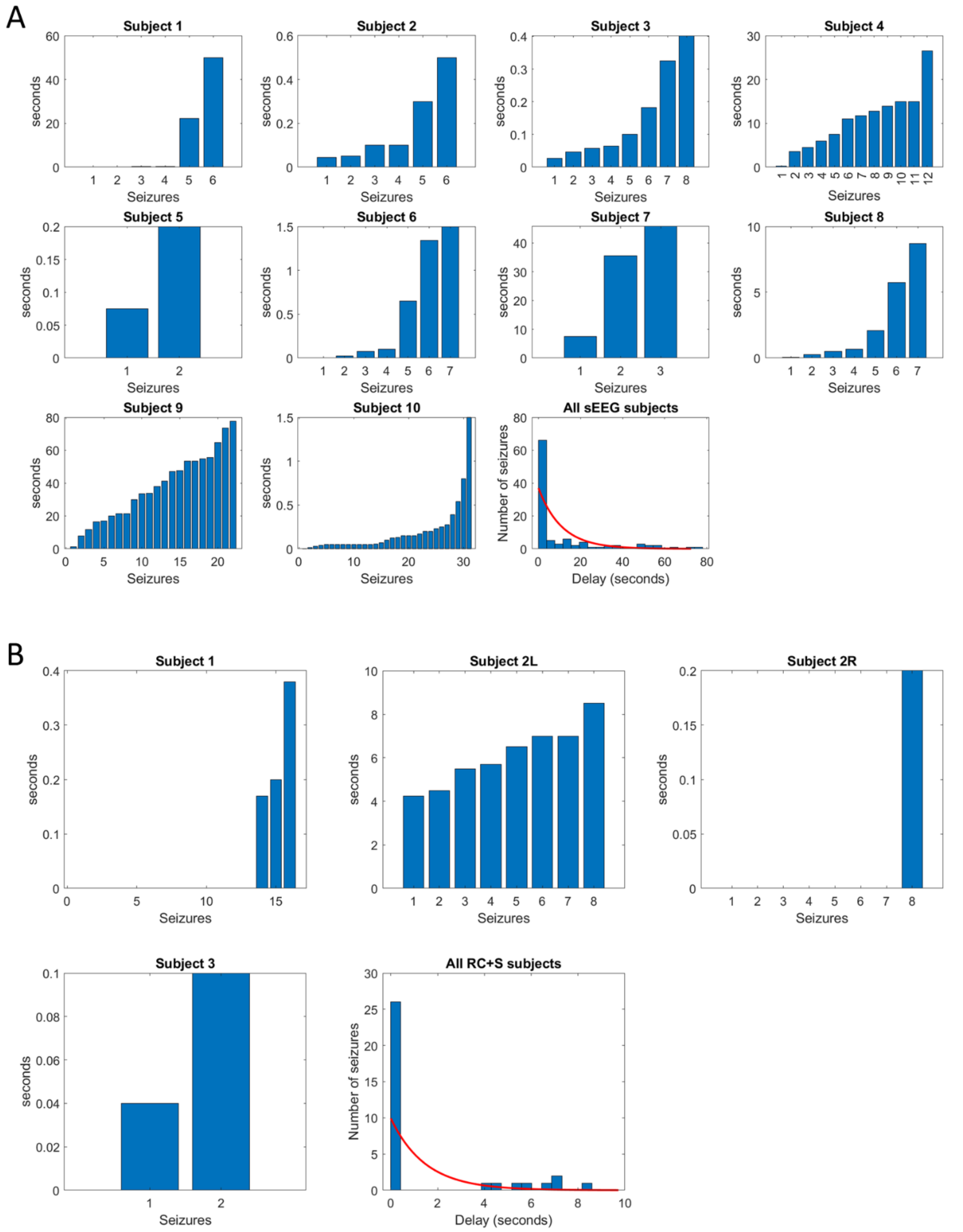

Supplementary Figure 1. Cortico-thalamic ictal discharge delays. (**A** & **B**) Delay distributions. Distributions of corticothalamic ictal onset delays are shown for each seizure in individual participants and averaged over all participants in **A** (stereo-electroencephalography [sEEG]) and **B** (RC+S^{TM}). Red line: fitted (exponential) histogram of corticothalamic delays.

Individual plots represent individual subjects, with the exception of subject RC+S2, for which each hemisphere is presented separately, as follows: ‘RC+S2R’ = Subject 2 right side, and ‘RC+S2L’ = Subject 2 left side. Each column in each plot represents a single seizure. Seizures are enumerated and ordered by duration from shortest to longest.

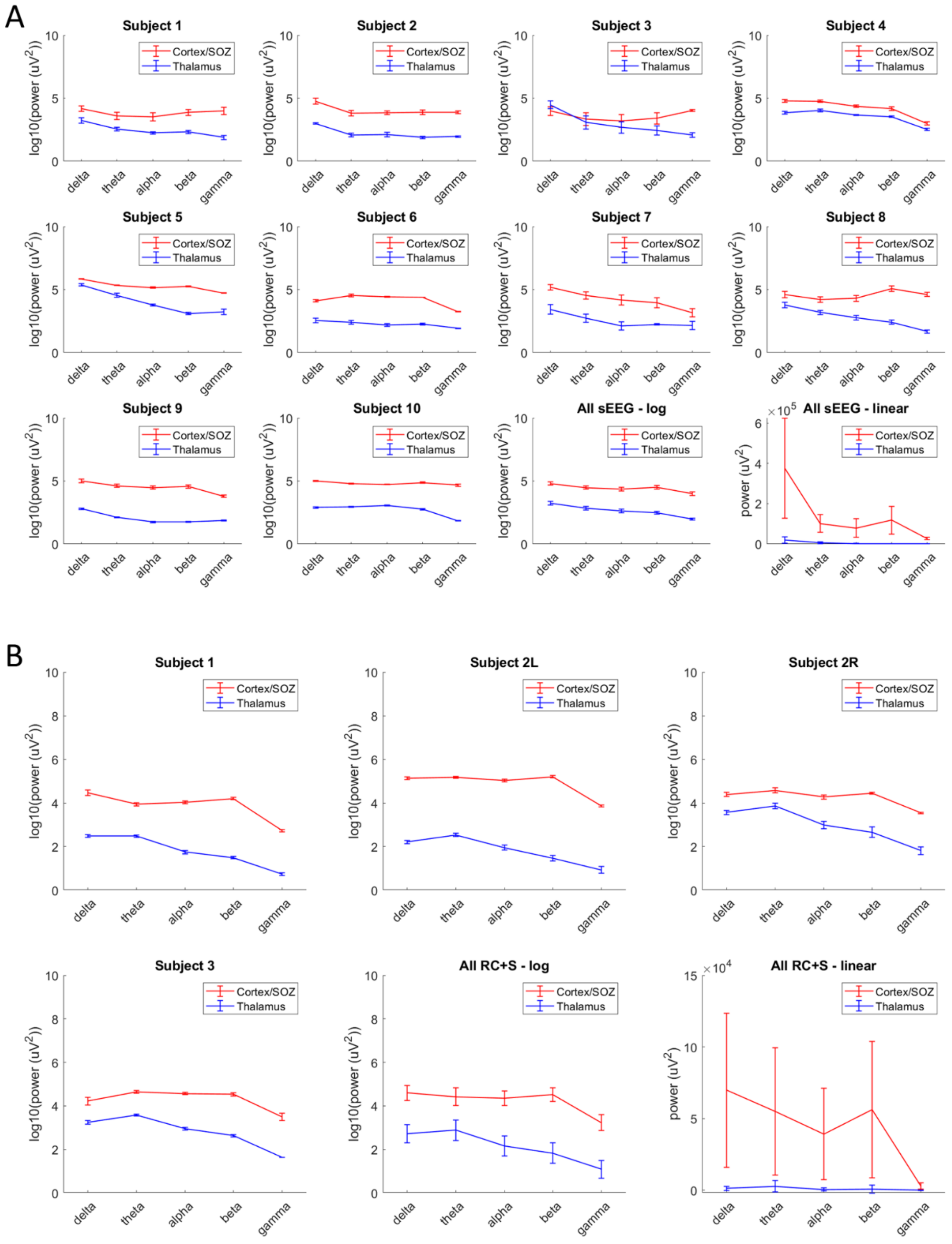

Supplementary Figure 2. Cortico-thalamic ictal discharge power differences. (**A** & **B**) Power in band in the cortical seizure onset zone (red), and thalamus (blue) are shown in log10 for each participant averaged across their seizures, and averaged across all participants in each category in **A** (stereo-electroencephalography [sEEG]) and **B** (RC+S^{TM}). Linear scale summary plots ("All sEEG – linear", "All RC+S – linear")) are provided along with log10

scale summary plots (“All sEEG – log”, “All RC+S – log”). Power in band is higher for all frequency bands on the group level and for most individuals, with the exception of lower (delta) frequencies in some participants. Frequency bands: delta = 0-4 Hz, theta = 4-8 Hz, alpha = 8-13 Hz, beta = 13-30 Hz, gamma = 30 – 100 Hz. Individual plots represent individual subjects, with the exception of subject RC+S2, for which each hemisphere is presented separately, as follows: ‘RC+S2R’ = Subject 2 right side, and ‘RC+S2L’ = Subject 2 left side. SOZ = seizure onset zone.

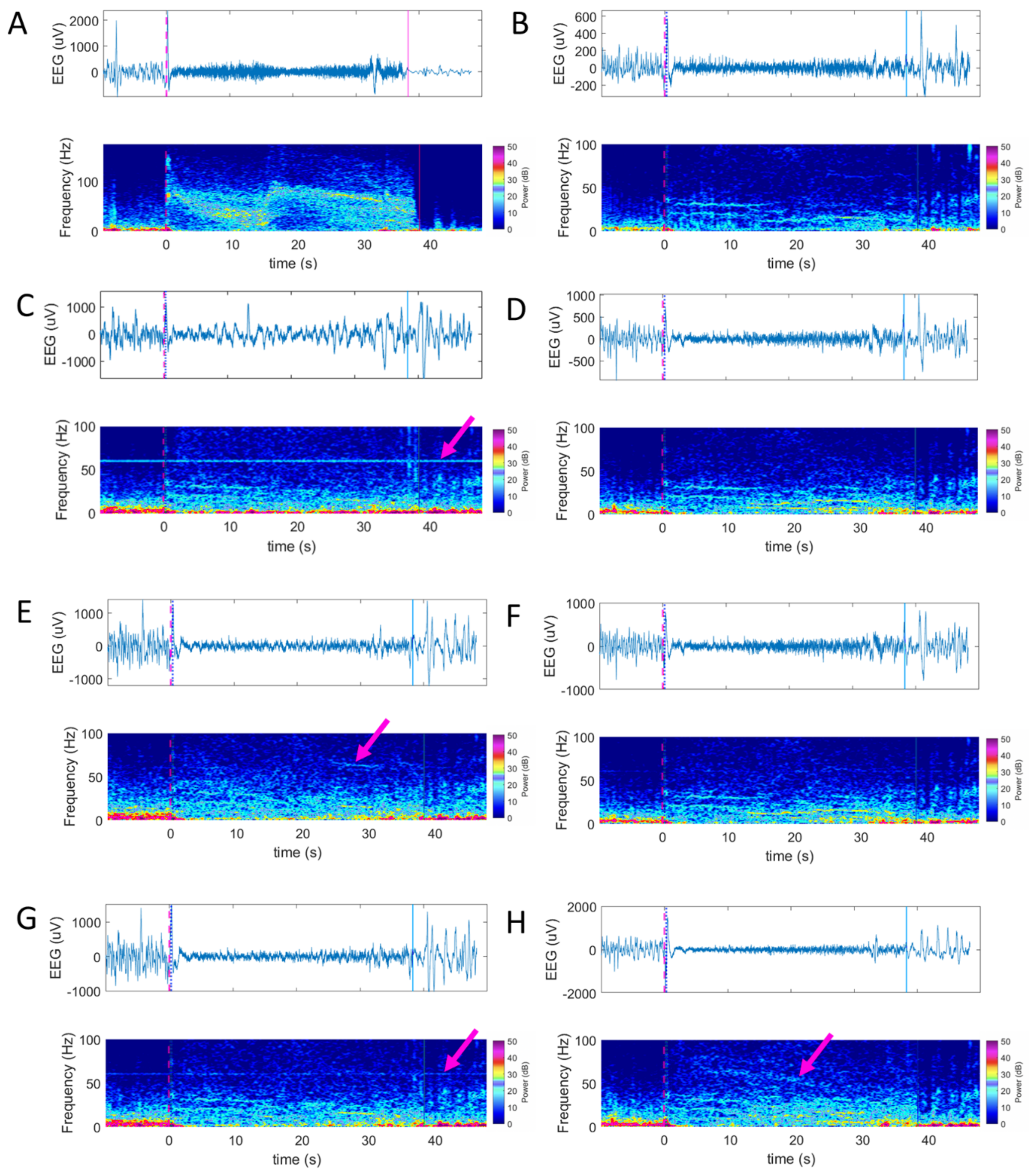

Supplementary Figure 3. Effects of re-referencing and alternative montages. The effects of re-referencing and alternative montages are shown for a single representative seizure from a single subject (same seizure in all panels). (**A & B**): Standard (alternate) bipolar montages of ictal discharge in the cortex (A) and thalamus (B). **(C-H):** effects of alternative montages including standard referential (scalp reference; C), re-referencing using white distant matter reference (D, F, H) or other contacts uninvolved in seizure onset on visual review (E, G). The primary organisation pattern of hypersynchrony (large spike) and low-voltage fast activity (best seen on spectrogram) is preserved in all montages. Artefact in the form of line noise (C, G; magenta arrows), and apparent volume conduction (E, H; magenta arrows), are more prominent in these montages.

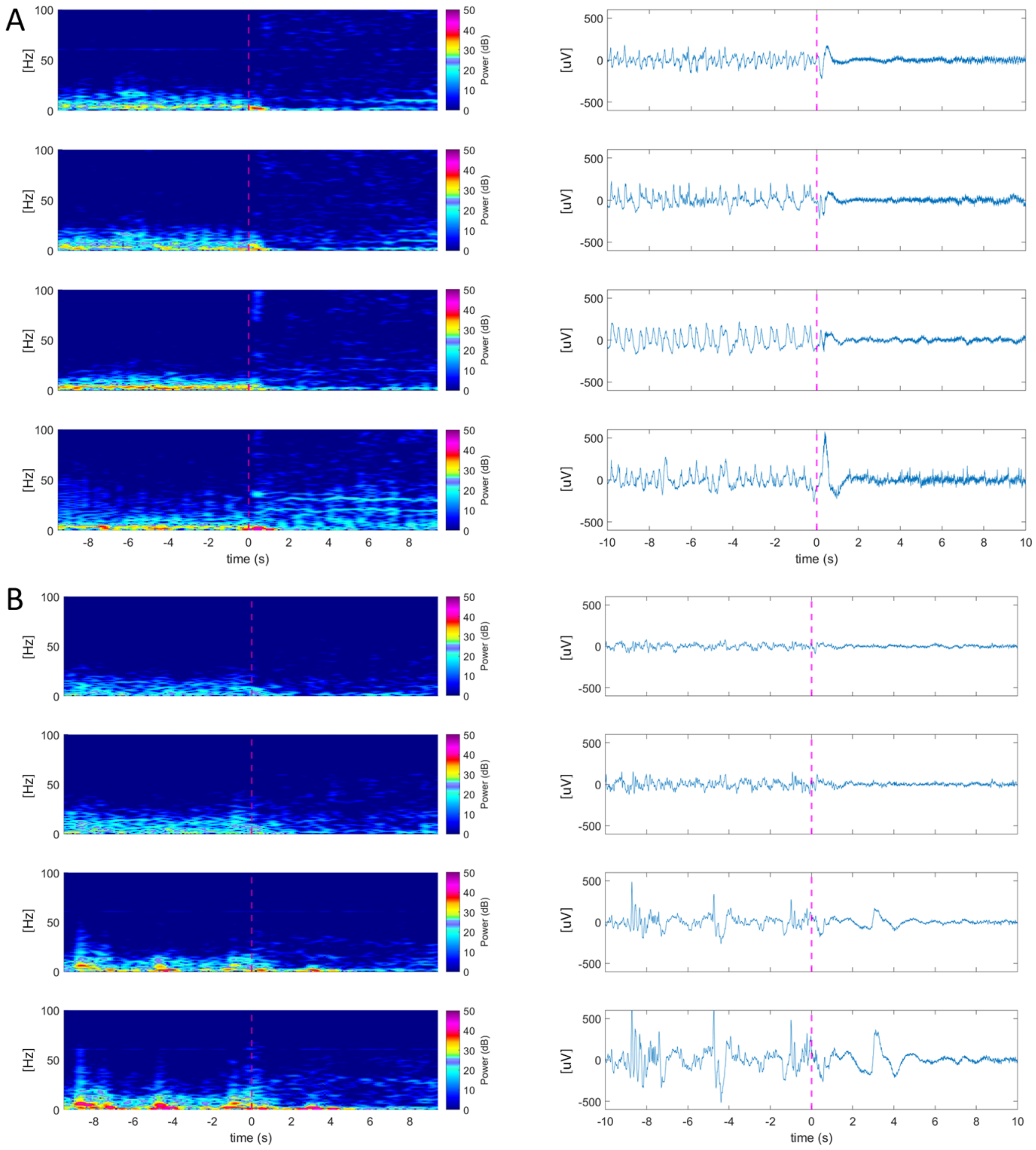

A
B
[Hz]
Power (dB)
[uV]
time (s)

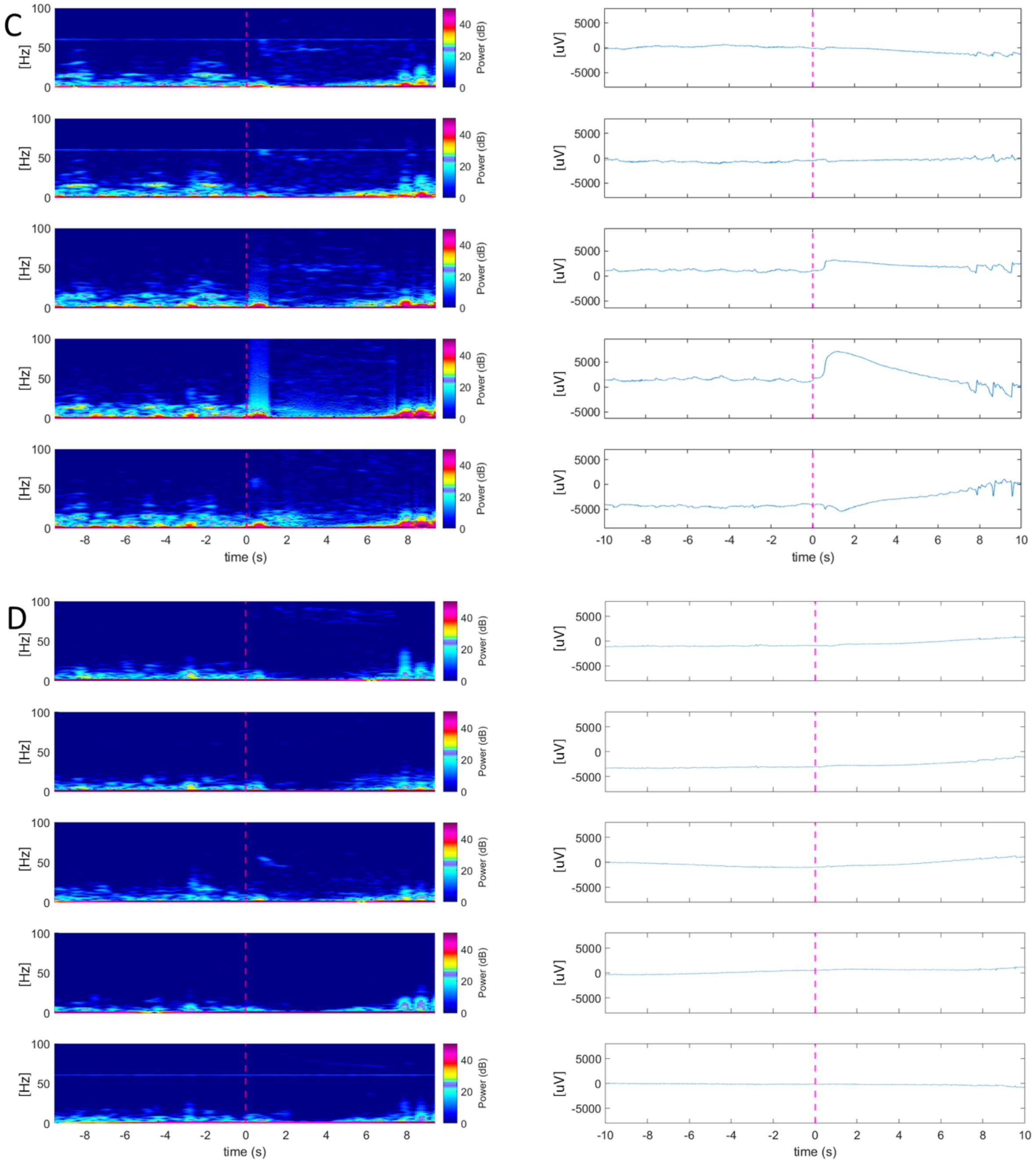

Supplementary Figure 4. Primary organisation patterns are specific to the thalamus. Two examples of the specificity of the primary organisation patterns are shown: a single seizure from one subject is shown in both A and B, while a single seizure from another subject is shown in both C and D. EEG from 10 seconds before and after ictal onset is presented for thalamic channels (A, C) and exemplar cortical channels not part of the seizure onset zone (B, D). **(A, B)**: For the seizure example presented in the main manuscript Fig 3C, thalamic channels demonstrate a large spike (hypersynchrony) and low voltage fast activity (low voltage fast activity [LVFA]; best appreciated in spectrogram); while these patterns are not observed in the uninvolved cortical channels (B). **(C, D)**: Similarly, for the seizure example presented in the main manuscript Fig 3D, thalamic channels demonstrate a large ictal baseline shift (direct current [DC]/baseline shift), and this pattern is not observed in the

uninvolved cortical channels (D). Note the y-axis (microvolts) scale differences required to appreciate the baseline shift.

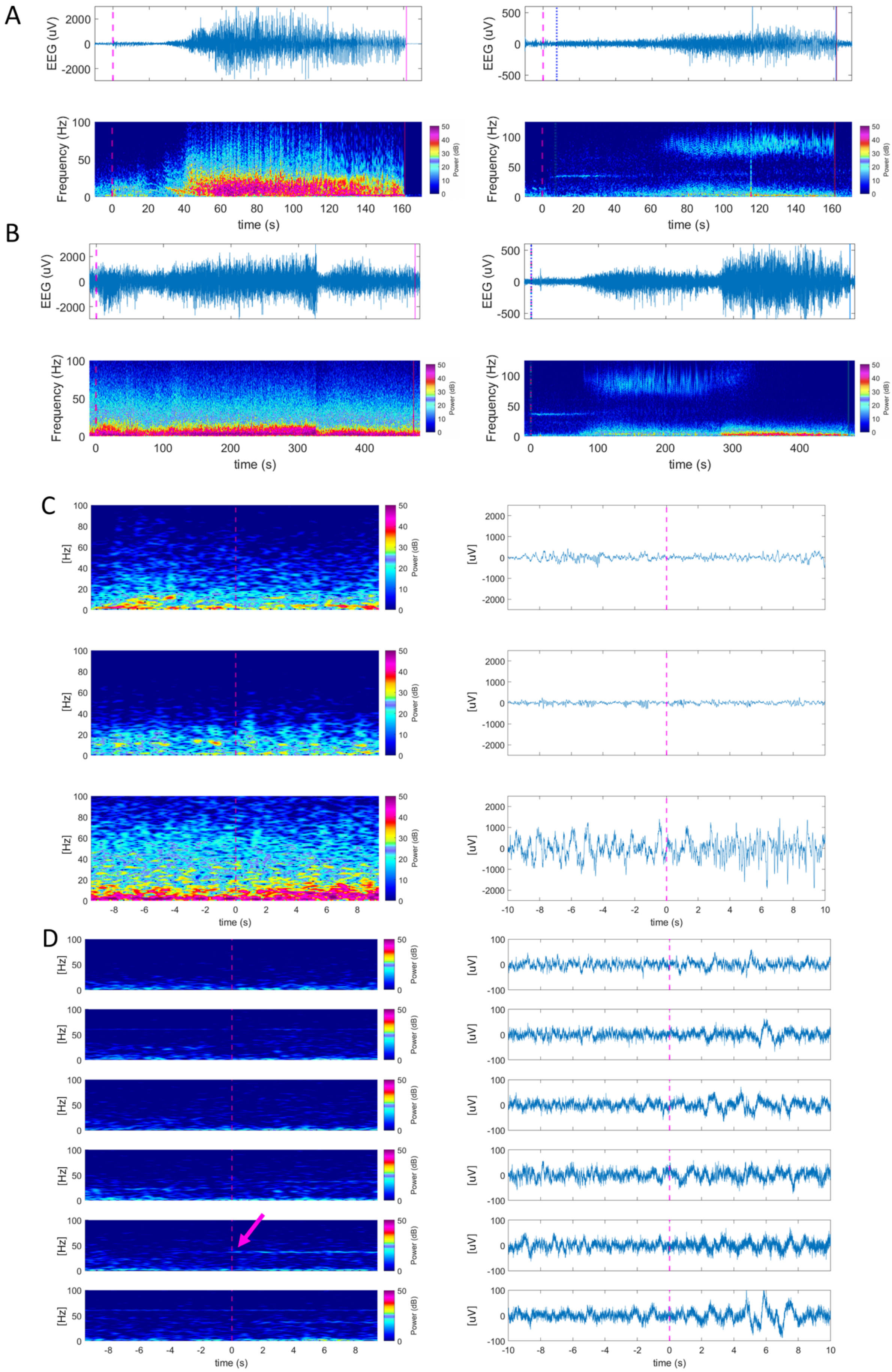
A
B
C
D
EEG (uV)
Frequency (Hz)
time (s)
Power (dB)
[Hz]
[uV]

Supplementary Figure 5. Example of thalamic discharge preceding cortical onset. (A & B): Two example seizures are shown from a single subject; one seizure is shown in A, and the second seizure is shown in B-D. Top left, cortical EEG; bottom left, cortical spectrogram; top right, thalamic EEG; bottom right, thalamic spectrogram. (A) In this seizure, a cortical seizure onset pattern is clearly seen, along with a thalamic propagation pattern which is delayed by several seconds. (B) In a subsequent seizure in the same patient, the same thalamic propagation pattern is seen without an obvious seizure onset pattern in the cortex. (C & D) The 10 seconds prior to and after the seizure shown in B for a few example cortical channels (C) and thalamic channels (D). A ~40 Hz thalamic discharge is notable (magenta arrow) preceding changes in the cortical channels by a few seconds.

Supplementary Table 1. Clinical characteristics.

Group / ID, Sex (M/F)	Age (yrs.) Epilepsy onset & implant	MRI Normal (N) Lesion (L)	sEEG / DBS implantation complications	Temporal vs extratemporal	Intervention performed (after sEEG/passive recording)	Sz reduction at last F/U	Length of F/U (months)
**sEEG**							
1 (M)	9; 22	R hippocampal T2 signal change w/o atrophy; then S/P R ATL	None	Extratemporal	LITT, then CSS (insular x2, ANT, CMT)	83%	55
2 (M)	7; 28	N	None	Extratemporal	None	N/A	1
3 (M)	9; 20	N	None	Extratemporal	Resection	86%	25
4 (F)	5; 35	N	None	Extratemporal	Neuromodulation (CSS; SMA, precentral x2, postcentral)	99%	83
5 (F)	11; 28	N	Lobar (parietal) hemorrhage (asymptomatic)	Extratemporal	Neuromodulation (CSS: ANT, 3 x peri-rolandic)	20%	87
6 (F)	59; 65	Increased T2 signal / volume L hippocampus / amygdala	None	Temporal	Neuromodulation (RNS L hippocampus)	93%	57
7 (F)	28; 30	N	None	Temporal & extratemporal	None	N/A	9
8 (M)	10; 33	L: left occipital encephalomalacia	None	Temporal & extratemporal	R HC LITT; L ANT + occipital RNS	62.5%	37
9 (F)	36; 39	L: Left MTS	None	Temporal	L HC LITT; L HC FUS; L HC LITT	82%	38
10 (F)	7; 47	N	None	Extratemporal	None	N/A	17
Median	9.5; 29						37.5
**RC+S™**							
1 (F)	9; 59	L: enlarged L amygdala / hippocampus	None	Temporal	ANT stimulation	80%	57
2 (F)	37; 45	N*#	None	Temporal	ANT stimulation	75%	45
3 (F)	4; 35	N*	None	Temporal	ANT stimulation	57%	41
Median	9; 45						45
Grand median	9; 33						41

*non-lesional MRI, but a history of anti-GAD antibody-associated autoimmunity is noted for these patients.

Vagus nerve stimulator (VNS) in situ prior to implant.

M = male; F = female; sEEG = stereo-EEG group; RC+S™ = investigational device group; DBS = deep brain stimulation; F/U = follow-up; L = left; R = right; N = normal; MTS = mesial temporal sclerosis; S/P = status post; ATL = anterior temporal lobectomy; LITT = laser interstitial thermal therapy; CSS = chronic/continuous subthreshold stimulation therapy; ANT = anterior nucleus of the thalamus; CMT = centromedian nucleus of the thalamus; SMA = supplementary motor area; RNS = responsive neurostimulation; HC = hippocampus/hippocampal; FUS = (high intensity) focused ultrasound; NA = not applicable/not available.

Supplementary Table 2. Seizure neurophysiology and semiology.

Group	Subj.	Temporal vs extra-temporal	Cortical seizure onset zone	Clinical manifestations	Thalamic discharge		Thalamic nucleus (grouping)	
					Presence: n (%)	Type: n (%) primary	First change	Maximal activation
sEEG	*1*							
		Extra-temporal	Anterior insula	None	2/2 (100)	0/2 (0)	Ventral anterior / lateral (VT)	Ventral anterior / lateral (VT)
		Extra-temporal	Posterior insula	Focal motor/FIAS	2/2 (100)	2/2 (100)	Centrolateral anterior (IT)	Ventral anterior / lateral (VT)
		Extra-temporal	Dorsolateral frontal (posterior orbital gyrus)	Focal motor/FIAS	2/2 (100)	2/2 (100)	Centrolateral anterior (IT)	Centrolateral anterior (IT)
	2							
		Extra-temporal	Posterior insula	Hypermotor (hyperkinetic)	6/6 (100)	4/6 (67)	Ventral lateral (VT)	Ventral lateral (VT)
	3							
		Extra-temporal	Frontal (dorsolateral)	Hypermotor	6/6 (100)	6/6 (100)	Mediodorsal (MT)	Pulvinar / lateral posterior (PT)
		Extra-temporal	Frontal (dorsolateral)	Hypermotor to bilateral tonic-clonic	2/2 (100)	2/2 (100)	Mediodorsal (MT)	Mediodorsal + centrolateral + pulvinar (MT/IT/PT)
	4							
		Extra-temporal	Frontal (precentral gyrus)	Subclinical / auras	12/12 (100)	0/12 (0)	Centrolateral anterior (IT)	Centrolateral anterior (IT)
			Frontal (precentral gyrus)	Hemiclonic	1/1 (100)	0/1 (0)	Centrolateral anterior (IT)	Centrolateral anterior (IT)
	5							
		Extra-temporal	Frontal (precentral gyrus)	Focal motor	2/2 (100)	2/2 (100)	Ventral lateral (VT)	Ventral lateral (VT)
	6							
		Temporal	Mesial temporal (amygdala)	Subclinical / minimally clinical	3/3 (100)	1/3 (33)	Ventral posterior lateral (VT)	Ventral posterior lateral (VT)
		Temporal	Mesial temporal (amygdala)	Aphasia	4/4 (100)	3/4 (75)	Ventral posterior lateral (VT)	Ventral posterior lateral + centrolateral + mediodorsal (IT/MT/VT)
	7							
		Temporal	Lateral temporal (ipsilateral)	Focal motor impaired awareness	1/1 (100)	0/1 (0)	Right ventral lateral / ventral posterior lateral (VT)	Right ventral lateral / ventral posterior lateral (VT)
		Temporal	Lateral temporal (contralateral)	Focal motor impaired awareness	2/2 (100)	0/2 (0)	Right centrolateral (IT)	Right ventral lateral (VT)
		Extra-temporal	Posterior insula (contralateral)	Focal motor	1/1 (100)	0/1 (0)	Right centrolateral (IT)	Right ventral lateral (VT)
	8	Extra-temporal	Parieto-occipital	Subclinical, visual auras	6/10 (60)	1/6 (17)	Pulvinar (PT)	Pulvinar (PT)
		Extra-temporal	Parieto-occipital	FIAS	1/1 (100)	0/1 (0)	Pulvinar (PT)	Pulvinar (PT)

		Temporal	Mesial temporal (contralateral)	FIAS	1/1 (100)	0/1 (0)	Pulvinar (PT)	Pulvinar (PT)
	9	Temporal	Mesial temporal	Subclinical or FAS (sensory)	22/36 (61)	0/22 (0)	Anterior nucleus (AT)	Anterior nucleus (AT)
	10	Extra-temporal	Dorsolateral frontal (middle frontal gyrus)	FIAS (behavioural / speech arrest, limb / trunk automatisms)	30/30 (100)	30/30 (100)	Ventral lateral (VT)	Ventral lateral (VT)
	Total				106/124 (85)	53/106 (50)		
							**Prior sEEG**	**Thalamus chronic implant**
RC+S^{TM}	*1*	Bitemporal L >> R	L mesial temporal<br><br>R mesial temporal	FIAS (behavioural arrest, arousal from sleep)<br><br>None recorded / analysed	16/16<br><br>NA	16/16<br><br>NA	Yes (no thalamic sampling)	L & R ANT
	2	Bitemporal L ~= R	L mesial temporal<br><br>R mesial temporal	Subclinical<br><br>FIAS (with automatisms)	8/8<br><br>8/8	0/8<br><br>8/8	Yes (no thalamic sampling)	L & R ANT
	3	Bitemporal L >> R	L mesial temporal<br><br>R mesial temporal	FIAS; behavioural/speech arrest oral/manual automatisms<br><br>None recorded/analysed	2/2<br><br>NA	2/2<br><br>NA	No prior sEEG	L & R ANT
	Total				34/34 (100)	26/34 (76)		
	Grand total				140/158 (89)	79/140 (56)		

M = male; F = female; sEEG = stereo-EEG group; RC+S^{TM} = investigational device group; DBS = deep brain stimulation; F/U = follow-up; L = left; R = right; AT = anterior thalamic group; VT = ventral thalamic group; IT = intralaminar thalamic group; MT = medial thalamic group; PT = posterior thalamic group; FAS = focal aware seizure; FIAS = focal impaired awareness seizure; ANT = anterior nucleus of the thalamus; HC = hippocampus/hippocampal; NA = not applicable/not available.

Supplementary Table 3. Thalamic sampling per subject and primary organisation pattern per thalamic region.

Subject		Thalamic location		Hypersynchronous			LVFA	Baseline / DC shift	Suppression
		**Group**	**Nucleus**	**Pre-ictal spiking**	**Sentinel spike**	**Repetitive spiking**			
sEEG	*1*	IT	CL	-	2/4	-	4/4	-	4/4
	2	VT	VL	-	-	-	4/4	-	-
	3	MT	MD	-	-	-	3/8	8/8	6/8
	4	IT	CL	-	-	-	-	-	-
	5	VT	VL	1/2	-	-	-	2/2	2/2
	6	VT	VL/VPL	-	-	-	4/4	4/4	4/4
	7	MT	CeM/MD	-	-	-	-	-	-
	8	PT	PuM	-	-	-	1/1	-	1/1
	9	AT	AV	-	-	-	-	-	-
	10	VT	VL	-	-	30/30	-	-	-
RC+S	*11*	AT	AV	8/16	-	-	16/16	-	16/16
	12	AT	AV	8/8	-	-	8/8	-	8/8
	13	AT	AV	-	-	-	2/2	-	2/2

For each subject, the number of times the specific onset pattern was noted is displayed (numerator) along with the number of seizures demonstrating any primary onset pattern (denominator), along with the region in which the primary organisation pattern was observed. AT = anterior thalamic group; VT = ventral thalamic group; IT = intralaminar thalamic group; MT = medial thalamic group; PT = posterior thalamic group; CL = centrolateral; VL = ventrolateral; MD = mediodorsal (dorsomedial); VPL = ventral posterior lateral; CeM = central medial; PuM = pulvinar, medial; AV = anteroventral; Y = yes.
